# GenSeq: An updated nomenclature and ranking for genetic sequences from type and non-type sources

**DOI:** 10.3897/zookeys.346.5753

**Published:** 2013-11-01

**Authors:** Prosanta Chakrabarty, Melanie Warren, Lawrence M. Page, Carole C. Baldwin

**Affiliations:** 1 Museum of Natural Science, Ichthyology Section, Department of Biological Sciences, Louisiana State University, 119 Foster Hall, Baton Rouge, LA USA; 2 Florida Museum of Natural History, University of Florida, Gainesville, FL USA; 3 National Museum of Natural History, Smithsonian Institution, Washington, DC USA

**Keywords:** GenBank, genetics, molecular phylogenetics, systematics, taxonomy

## Abstract

An improved and expanded nomenclature for genetic sequences is introduced that corresponds with a ranking of the reliability of the taxonomic identification of the source specimens. This nomenclature is an advancement of the “Genetypes” naming system, which some have been reluctant to adopt because of the use of the “type” suffix in the terminology. In the new nomenclature, genetic sequences are labeled “genseq,” followed by a reliability ranking (e.g., 1 if the sequence is from a primary type), followed by the name of the genes from which the sequences were derived (e.g., genseq-1 16S, COI). The numbered suffix provides an indication of the likely reliability of taxonomic identification of the voucher. Included in this ranking system, in descending order of taxonomic reliability, are the following: sequences from primary types – “genseq-1,” secondary types – “genseq-2,” collection-vouchered topotypes – “genseq-3,” collection-vouchered non-types – “genseq-4,” and non-types that lack specimen vouchers but have photo vouchers – “genseq-5.” To demonstrate use of the new nomenclature, we review recently published new-species descriptions in the ichthyological literature that include DNA data and apply the GenSeq nomenclature to sequences referenced in those publications. We encourage authors to adopt the GenSeq nomenclature (note capital “G” and “S” when referring to the nomenclatural program) to provide a searchable tag (e.g., “genseq”; note lowercase “g” and “s” when referring to sequences) for genetic sequences from types and other vouchered specimens. Use of the new nomenclature and ranking system will improve integration of molecular phylogenetics and biological taxonomy and enhance the ability of researchers to assess the reliability of sequence data. We further encourage authors to update sequence information on databases such as GenBank whenever nomenclatural changes are made.

## Introduction

The use of genetic sequences has been increasing with each passing year (Benson et al. 2005; Strasser 2011); unfortunately, the separation between voucher specimens and genetic sequences is similarly growing. With increasing frequency, the link between the genetic sequences being used in analyses and the organisms from which they came is not being reported (Pleijel et al. 2008). GenBank and other depositories are excellent sources of genetic sequences that have a strong system for accurately identifying genetic data being submitted (e.g., COI cannot be mislabeled as 16S), but little is done to check the accuracy of the identity of the organism from which the sequences were obtained (Federhen et al. 2009). The taxonomic determination remains solely the responsibility of the submitter of the sequences. Erroneous identifications are difficult to discover, and the perpetuation of the error in subsequent uses of the sequence data is nearly impossible to stop. Once a sequence is published, the identification rarely is questioned unless another sequence from the same gene and species is noted to be substantially different, or sequences from putatively unrelated taxa are very similar (e.g., Baldwin et al. 2009). Likewise, an identification may be questioned if a BLAST (*Basic Local Alignment Search Tool*, in GenBank) search or phylogenetic analysis reveals a sequence to be in an unexpected region of a similar species or in an unexpected part of a phylogeny.

Although an institutional catalog number for the specimen (the ‘voucher’) from which a sequence is obtained is often requested when a sequence is submitted, it is not obligatory. Most sequences available on GenBank lack this information. Sequences available from databases such as GenBank have little reference to the source of the genetic materials other than the title and authors of the original publication. Unfortunately, original publications also often lack information about the original specimens necessary to validate their identification. To remedy this deficiency and to remind researchers about the importance of providing and accurately identifying DNA voucher specimens, we propose a new genetic nomenclature based on a ranking of various source specimens. We also suggest various ways in which the link between specimens and genetic sequences can be made more transparent.

Chakrabarty (2010) proposed the ‘Genetypes’ nomenclature to help flag genetic materials from type specimens in scientific papers and other outlets. This classification allowed researchers to more readily find sequences from type specimens where there is certainty that a specimen was vouchered and little doubt (none for primary types) that the voucher was correctly identified. The unfortunate use of the word “type” in the Genetypes nomenclature (e.g., “hologenetype” for sequences from a holotype) led some to think that the sequences were being designated as representative genetic types for the species just as type specimens are. That was not the intention; rather, the intent was to emphasize the reliability of those sequences because of the reliable taxonomic identification associated with type specimens. The sequences themselves are not unique identifiers (name-bearers) of the species, and the “type” suffix is not included in the new GenSeq nomenclature. The goal of the new nomenclature remains the same as that of the original: to assess the reliability of sequence data by increasing the transparency of links between specimens, taxonomy, sequence data, and molecular evolutionary analyses.

The GenSeq nomenclature combines the term “genseq” with a hyphen and a number from 1 to 5 reflecting the reliability ranking we provide in Table 1. Sequences from primary type specimens are referred to as genseq-1, with the 1 reflecting the highest reliability rank. In addition to these terms, the gene region(s) should be reported with the GenSeq reference; for example, “genseq-1 mitogenome, genseq-2 16S, ND2”, or “genseq-5 UCE chr11_2436”. The hyphen between “genseq” and the number must be included to allow search engines such as Google to search the entire text string because these searches treat hyphens as spaces. Note that in reference to the nomenclatural program we use capital letter “G” and “S” (e.g., GenSeq), and all lowercase letters (e.g., genseq-1) when referring to specific sequences.

**Table 1.** Ranking Sequence Reliability. Ranking of source materials of genetic sequences based on reliability of taxonomic identification. Examples of the source material are listed in the third column with the last column providing the corresponding GenSeq nomenclature.

Reliability Ranking	Source Materials	Examples	Corresponding GenSeq Nomenclature
Highest 1^ST^	Primary Types	Holotype, Lectotype, Syntype, Isosyntype, Neotype, Isotype	genseq-1
2^nd^	Secondary Types	Paratype, Paralectotypes, etc.	genseq-2
3^rd^	Topotypes (vouchered), or non-type specimens listed in original description or redescription	Topotype, Non-type specimen listed in original description or redescription	genseq-3
4^th^	Collections-vouchered non-types (not from original description or redescription)	Vouchered specimen	genseq-4
5^th^	Photo voucher only	No specimen voucher but photo voucher available	genseq-5
Lowest	No voucher	Non-vouchered	No classification

**Figure 1.**
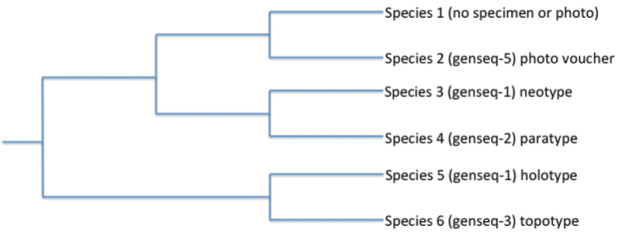
Example of how the GenSeq ranking system of sequences from various sources (Table 1) can be used to assess the trustworthiness of data used to reconstruct phylogenetic relationships. The rankings (the # in the “genseq-#”) make it clear that the relationship recovered between Species 3 and Species 4, from primary and secondary types, should be trustworthy because the taxonomic identifications of the voucher specimens are considered to be highly reliable. In contrast, the recovered sister relationship between Species 1 and Species 2 may be less trustworthy because of the weak reliability rankings of these sequences from non-types. Species 1 lacks both a specimen or photo voucher and therefore does not have a GenSeq ranking.

Many GenSeqs can be created from a single specimen and can be from a single gene fragment, multiple fragments, or an entire genome; for instance, “genseq-2 COI” and “genseq-2 COII, ND2,” could be added from the same paratype voucher at a later date, as could GenSeqs from other specimens of this species (e.g., other paratypes, the holotype) from which DNA was extracted. This nomenclature is simply a flag to alert molecular biologists and taxonomists that sequences are available from type specimens and some confidently identified non-types (see below). We suggest that researchers preferentially use these sequences in molecular evolutionary analyses, as doing so should bolster confidence in conclusions based on the sequence data (Fig. 1). Tabulating the GenSeq nomenclature with GenBank numbers and catalog numbers for vouchers will provide subsequent workers with easy access to this information (Table 2). We suggest that authors report the GenSeq in either the *Systematic Accounts* section or *Materials and Methods* section of a manuscript. For these sections, an additional example of how the GenSeq nomenclature could be reported for type specimens is: “One of the paratypes (USNM 139024) was sequenced (GenBank accession number JZ254935) and therefore constitutes a genseq-2 cytochrome *b*.”

**Table 2.** Example Reporting Table. Examples of how links between genetic sequences and vouchers in institutional collections could be displayed as a table in publications reporting new sequences.

Species	Specimen Catalog #	GenBank #		GenSeq Nomenclature
		COI	ND1	
*Typhleotris mararybe*	LSUMZ 13636 (a holotype)	HM590594	HM590606	genseq-1 COI, ND1
*Paretroplus tsimoly*	AMNH 229558 (a paratype)	JZ590596	NA	genseq-2 COI
*Nandopsis haitiensis*	UMMZ 236321 (a topotype)	BK590595	BK590607	genseq-3 COI, ND1
*Halieutichthys intermedius*	FMNH 96353 (a non-type specimen voucher)	AY722169	AY722306	genseq-4 COI, ND1
*Equulites absconditus*	NMNH 12345PV2 (a photo voucher)	NA	BG34621	genseq-5 ND1

The “genseq” suffix will flag genetic sequences in any manuscript so that authors can better assess the reliability of the taxonomic identification of specimens used to obtain those sequences. A sequence from a holotype provides the highest reliability of taxonomic identification and is therefore awarded the highest ranking in the system (genseq-1; Table 1). Although secondary types are not name-bearing specimens, in most cases identifications of secondary types are at least as trustworthy, and generally more so, than those from non-types, which may have been identified by non-experts or that may lack vouchered specimens. For this reason, sequences from secondary types are given the second highest ranking (genseq-2). Sequences from vouchered topotypes—individuals collected from the type locality of a species—are given the third highest ranking as are individuals not designated as types in the original description but are identified as belonging to the new taxon in that same manuscript (both genseq-3).

The genseq-3, -4, and -5 categories (Table 1) represent a departure and expansion from the previous Genetypes nomenclature. Because most species included in molecular analyses will not have type specimens available for sequencing, it is important to expand the genetic nomenclature and ranking to forecast the reliability of other sources of sequences. A sequence from a vouchered specimen that was not included in the type series but that is identified in the original description of the species as a member of the new taxon should be flagged as “genseq-3.” The “3” is used as a suffix in this case again to reflect that it belongs to the third highest category of reliability (Table 1). Sequences from vouchered specimens from a redescription of a resurrected species should also be flagged “genseq-3.” Sequences from non-type specimens that are not mentioned in the original description, or redescription, but that are confidently identified by an expert should be flagged “genseq-4” with the “4” suffix again reflecting the 4^th^ highest ranking of reliability. Finally, “genseq-5” is a flag for sequences that lack any specimen voucher but that have a well-documented and publically available photo voucher. A photo voucher is not ideal but is necessary when the organism is still alive, highly endangered, extremely large, or extremely small (e.g., a larva where the entire sample must be used to obtain sufficient DNA).

In cases where the term “genseq-[3, 4, or 5]” category is used to identify sequences that are from a non-type specimen, the specimen should be identified by an authority and deposited in a reputable natural history collection. Both specimen and photo vouchers would be included in the “hologenophore” category of biological vouchers described by Pleijel et al. (2008: 369), which indicates that the voucher is the same individual organism from which (in molecular biology) the genetic data were derived. As the GenSeq nomenclature applies only to hologenophores, the remaining categories of biological vouchers of Pleijel et al. (2008), in which the voucher is not the same individual from which (in molecular biology) the genetic sequences were derived, are not relevant to the GenSeq nomenclature.

To better understand how sequences from type specimens are currently being reported in the scientific literature, we conducted a survey of recent publications describing new species of fishes that included DNA data. Fishes were chosen in part because we are ichthyologists, but also because fishes are described at a rate that is the highest among vertebrates (Lundberg et al. 2000). After tabulating new species from the relevant publications, we apply the GenSeq nomenclature to sequences referenced in those publications.

## Methods

We used a Google Scholar search (www.scholar.google.com) to find papers published between 2010 and 2011 using the search term “new fish species DNA”. The retrieved papers were reviewed for any mention of sequences obtained from a holotype or paratypes. Because many papers did not link the GenBank #’s with the voucher’s catalog number, we conducted a corresponding search on GenBank (http://www.ncbi.nlm.nih.gov/) to determine whether those catalog numbers were reported there.

Each species recovered from the Google Scholar search was entered into the GenBank “nucleotide” search-engine field. If a catalog number of a holotype or paratype(s) was recovered in either the original paper or with GenBank, it was reported in Table 3. In cases where the link between voucher and sequence was unclear, authors of the descriptions were contacted to clarify the link. We only report examples where the genetic sequences could be positively linked to the catalog number of the holotype or paratypes. (Other forms of GenSeqs were not searched for in this initial case study.)

**Table 3.** Results of Search for Sequences from Types. GenSeq nomenclature applied to DNA sequences of fishes described from 2010–2011. The data were mined from GenBank and Google Scholar. Institutional abbreviations follow Sabaj-Perez (2012) except GSDNA which is the Natural History Gallery of Casalina. ◆ indicates that the catalog number of the voucher was reported with the genetic sequences in the published original description. ○ indicates that the catalog number of the voucher was recorded in GenBank with the sequences. Lack of either symbol indicates that the authors were e-mailed to find the link between a voucher and a sequence.

Species (Group)	Citation	Type of type	Voucher catalog	GenBank #	GenSeq
*Bathygobius antilliensis* (Teleostei: Gobiidae)	Tornabene et al. 2010 ◆○	Holotype	AMNH 251650	HM748393	genseq-1 COI
		Paratypes	16 examples	16 examples	genseq-2 COI
*Bathygobius geminatus* (Teleostei: Gobiidae)	Tornabene et al. 2010 ◆○	Holotype	USNM 398105	HM748368	genseq-1 COI
		Paratypes	AMNH 251648	HM748389	genseq-2 COI
			USNM 398102	HM748357	
			USNM 398103	HM748365	
			USNM 398106	HM748369	
			USNM 398107	HM748373	
			USNM 398109	HM748375	
			USNM 398112	HM748379	
*Chimaera opalescens* (Chondrichthyes: Holocephali)	Luchetti et al. 2011 ◆○	Paratypes	MNHN 2007-1557	GU244532	genseq-2 COI
			MNHN:2007-1555	GU244533	
			MNHN:2007-1567	GU244534	
			MNHN:2007-1579	GU244531	
*Callopanchax sidibei* (Nothobranchiidae: Epiplateinae)	Sonnenberg and Busch 2010	Holotype	ZFMK 41613	GU553012	genseq-1 16S
*Hypsolebias guanambi* (Cyprinodontiformes: Rivulidae)	Costa et al. 2011 ◆○	Paratypes	UFRJ 6782.1	HQ833483	genseq-2 CytB
			UFRJ 6782.2	HQ833484	
			UFRJ 6782.3	HQ833485	
			UFRJ 6782.4	HQ833486	
*Petrocephalus similis* (Osteoglossomorpha: Mormyridae)	Lavoue 2011 ○	Holotype	CU 95318	JF438961	genseq-1 CytB
		Paratypes	CU 93218.1	JF438960	genseq-2 CytB
			CU 93218.2	JF438962	
			CU 93218.3	JF438964	
			CU 93218.4	JF438963	
			CU 93219	JF438965	
*Starksia sangreyae* (Teleostei: Labrisomidae)	Baldwin et al. 2011 ◆	Holotype	USNM 398932	HQ600872	genseq-1 COI
		Paratypes	USNM 398939	HQ600865	genseq-2 COI
			USNM 398933	HQ600873	
			USNM 398936	HQ600868	
			USNM 398934	HQ600875	
			USNM 398935	HQ600874	
			USNM 398938	HQ600866	
			USNM 398940	HQ600864	
*Starksia springeri* (Teleostei: Labrisomidae)	Baldwin et al. 2011 ◆	Paratypes	USNM 399658	HQ600878	genseq-2 COI
			USNM 399659	HQ600876	
*Starksia weigti* (Teleostei: Labrisomidae)	Baldwin et al. 2011 ◆	Paratypes	USNM 399649	HQ600886	genseq-2 COI
			USNM 399653	HQ600934	
			USNM 399652	HQ600935	
			USNM 399651	HQ600936	
			USNM 399656	HQ600927	
			USNM 399655	HQ600932	
*Starksia williamsi* (Teleostei: Labrisomidae)	Baldwin et al. 2011 ◆	Paratypes	USNM 397396	HQ543039	genseq-2 COI
*Starksia robertsoni* (Teleostei: Labrisomidae)	Baldwin et al. 2011 ◆	Paratypes	USNM 399909	HQ600961	genseq-2 COI
			USNM 399911	HQ600960	
*Starksia greenfieldi* (Teleostei: Labrisomidae)	Baldwin et al. 2011 ◆	Paratypes, Topotypes	USNM 398922	HQ600924	genseq-2 COI, genseq-3 COI
			USNM 398921	HQ600925	
			USNM 398920	HQ600947	
*Esox flaviae* (Esociformes, Esocidae)	Lucentini et al. 2011 ◆	Holotype	GSDNA1	HM563688.1 (COI)	genseq-1 COI, CytB
				JN190460 (cytB)	
*Milyeringa brooksi* (Teleostei: Gobiiformes)	Chakrabarty 2010 ◆○	Paratype	LSUMZ 13637	HM590607 (ND2), HM590601 (cytB), HM590595 (COI)	genseq-2 ND2, CytB, COI
*Leptoderma macrophthalmum* (Otocephala: Alepocephalidae)	Byrkjedal et al. 2011 ◆	Holotype	ZMUB 19686	AP011500	genseq-1 mitogenome
*Sparisoma rocha* (Actinopterygii: Labridae)	Pinheiro et al. 2010	Paratype	ZUEC 6349	GU985520 (16S)	genseq-2 16S, 12S
				GU985521 (12S)	
*Halichoeres rubrovirens* (Perciformes: Labridae)	Rocha et al. 2010 ○	Paratype	CIUFES 0317	GU938858	genseq-2 CytB
			CIUFES 1279	GU938859	
			CIUFES 1474	GU938860	
			CIUFES 1475	GU938861	
			USNM 397005	GU938862	
*Betadevario ramachandrani* (Cyprinidae: Danioninae)	Pramod et al. 2010 ◆	Paratype	NRM 57780	GU327623 (cytB), GU327622 (Rho)	genseq-2 CytB, rhodopsin
*Crenicichla hu* (Teleostei: Cichlidae)	Piálek et al. 2010 ◆	Paratype	MACN-ict 9430.1	GQ328038 (trnQ, trnM, ND2, trnW, trnA)	genseq-2 trnQ, trnM, ND2, trnW, trnA
			MACN-ict 9430.2	GQ328039 (trnQ, trnM, ND2, trnW, trnA)	

## Results

The Google Scholar search produced 47 publications from 2010 and 2011 that included descriptions of new species of fishes and used sequence data. Only 13 of those papers indicated that sequences were derived from a type or non-type specimen (Table 3). Of the remaining 34 publications there was either no clear link between catalog numbers of vouchers and sequences (even after a query e-mail was sent to a corresponding author), or, rarely, it was made clear in the paper that no types were among those sequenced.

Of the 13 publications in Table 3, only three reported the catalog number of the type specimens along with the GenBank #’s, both in the manuscript and on GenBank. Six others reported both numbers only in the paper, and two reported the catalog number solely on GenBank. The two remaining papers (of the 13) were verified to have sequences from a primary or secondary type only after a query e-mail to the corresponding author. These authors did not supply the catalog numbers of the voucher specimens from which GenBank sequences were obtained either in their manuscript or on GenBank.

This is not a complete list of descriptions of new species of fishes with genetic sequences from type specimens. There are likely some publications that were not found via GoogleScholar or that would have been found in other search engines because of the nature of the scripts used in those searches. This search on descriptions of new species of fishes using sequence data is only a rough proxy for other groups of organisms.

## Discussion

GenSeq is a nomenclatural label for sequence data from confidently identified vouchered specimens. By explicitly flagging gene sequences from type materials, the new nomenclature will enable researchers to utilize sequences from the best-identified specimens when available. In particular, “genseq-1” and “genseq-2” flags will highlight sequences (see Table 1) from GenBank that, due to their direct link to primary and secondary type specimens, will be more credible than sequences from specimens with less certain identifications. Type materials remain essential for taxonomic comparisons, but sequence data from type materials have not been fully incorporated into these comparisons (see references in Chakrabarty 2010; present study).

The burden of linking specimens (even type specimens) to sequences from the publications of others is one reason for the creation of this expanded nomenclature. Authors often do not provide a clear link between voucher specimens and the sequences obtained from them. Presumably authors publishing on taxonomy and molecular phylogenetics would be much better at providing a clear link between the two, but as made evident from the results of this study, these authors often fail to make this link. Unfortunately, many authors simply make a statement similar to the following: “the sequences obtained from this study were given the GenBank #’s XX12428-XX12531,” which tells no one which sequence belongs to which specimen (or even which species). These data are even more poorly reported on GenBank, where few researchers provide catalog numbers of the vouchers from which the sequences were obtained. A clear link between the specimens’ catalog number and the sequences’ GenBank #’s should either be made in a table (as in Table 2), or the voucher’s catalog numbers should be listed in GenBank with the genetic sequences. Ideally, both tasks should be done to maximize transparency.

The GenSeq nomenclature also incorporates sequences from non-type materials because many species will never have their type specimens sequenced. This could be because some collections will not want the morphological integrity of the type specimen to be diminished by the removal of a subsample for DNA extraction, or because the specimen has been fixed in formalin (as is the case for most fish, reptile, and amphibian specimens), or by some other preservation method and will no longer yield sufficient amounts of DNA. An example of how one of the non-type flags (viz., genseq-3, genseq-4, genseq-5) can be used is in cases of a taxonomic treatment in which a formerly synonymized species is resurrected and there is not a type specimen that will yield DNA. Sequences from a fresh specimen for the resurrected species should be flagged as “genseq-3.” Because the identification of the voucher is tied to the work of a taxonomist resurrecting a species (i.e., the first reviser), other researchers should have high confidence in its correct identification. Although not type material, such specimens and sequences from them should be regarded as highly likely to be correctly identified.

A Google Scholar search of the usage of the former Genetypes nomenclature, revealed 24 citations from 2010–2013 for sponges, fungi, fishes, amphibians, birds, mammals, and insects. Without a search term (in this case Genetypes), finding sequences that are derived from type specimens requires reading original publications or looking up sequences in a database. As our results indicate, authors are often inconsistent in how this information is reported, if they choose to report it at all. The benefits of having a search term like “genseq-#” embedded in a manuscript can be demonstrated by doing a simple Google Scholar search on a similar label, such as “holotype;” such a search can be rendered even more specific by adding a scientific name, e.g., “holotype *Typhleotris mararybe*.” Our new genetic terminology will enable researchers to conduct searches such as “genseq-# + *Genus species*,” which will help them locate genetic sequences from well-documented, and likely properly identified, vouchered specimens.

Ultimately, the GenSeq approach will benefit all forms of taxonomic research as molecular phylogenetics becomes integrated with taxonomy and as technology improves in molecular biology. We remind researchers about the importance of vouchers and reporting taxonomic changes to databases such as GenBank. Taxonomic changes, misidentifications, and other changes to sequences need to be reported before they are perpetuated erroneously in the literature. If a species has sequences on GenBank and that species is later split into two species, the taxonomy should be updated by the authors on GenBank. Without this update, the original GenBank sequences that represent the new species in the split, rather than the existing one, may be used erroneously by unsuspecting researchers.

To expand usage of GenSeq flags for genetic sequences, a summary of this nomenclature should be incorporated into the “Instruction to Authors” for taxonomic journals. Harrison et al.’s (2011) editorial was used to explain the usage of the Genetypes nomenclature to authors using the *Journal of Fish Biology*. We suggest that the following text be added to the author guidelines of taxonomic journals where sequences are reported:

**Sequence data:** Manuscripts containing novel amino acid sequences (e.g. primer sequences) will only be accepted if they carry an International Nucleotide Sequence Databases (INSD) accession number from the European Biology Laboratory (EMBL), GenBank Data Libraries (GenBank) or DNA Data Bank of Japan (DDBJ). [Name of Journal] strongly recommends that authors include institutional catalog numbers for specimens preserved in collections, and information identifying sequences that are derived from type specimens (see below) when they deposit data in genetic databanks. Database [GenBank] accession [catalog] numbers should be included in the Materials and Methods section. If specimens were not vouchered (tissued specimens should be vouchered when possible!), photographs and collection locality data for tissued specimens must be provided.

A nomenclature for genetic sequences for types and confidently identified non-type specimens has been proposed by Chakrabarty et al. (2013); a sequence from a holotype is identified as genseq-1, one from a paratype is identified as genseq-2, one from a topotype is genseq-3, etc. The genetic marker(s) used should also be incorporated into the nomenclature (e.g. genseq-2 COI).

Authors who wish to report GenSeqs in a web interface (in addition to in a published manuscript and on GenBank) may choose the widely used Animal Diversity Web (ADW; http://animaldiversity.ummz.umich.edu/). The editors of this website suggest that the “Other Comments” field can be used to report GenBank links of GenSeqs (Tanya Dewey pers. comm.). For help creating an ADW page for a new taxon using the GenSeq nomenclature, please contact the first author of this paper.

In the future, we hope the GenSeq nomenclature will be widely used and eventually incorporated into GenBank and other large genetic databases. Incorporation is not currently possible with GenBank’s user-driven interface which would allow too much human error in labeling sequences (pers. com., Scott Federhen, GenBank; Federhen et al. 2009). To be more specific, a user may apply the label “genseq” erroneously, and there is currently no accuracy-checking system within GenBank to correct that error. Our hope is that usage of this nomenclature will increase the rigor of evolutionary analyses using molecular sequences and remind authors to provide a clear link between sequences and vouchers.
